# Transcriptomic and metabolomic analyses for the mechanism underlying anthocyanin synthesis during the growth and development of purple eggplant (*Solanum melongena* L)

**DOI:** 10.3389/fpls.2025.1577367

**Published:** 2025-05-15

**Authors:** Ting Yang, Wanping Lv, Runjie Zhuang, Shiyu Wang, Shuqi Fan, Yongxian Wen

**Affiliations:** ^1^ College of Computer and Information Science, Fujian Agriculture and Forestry University, Fuzhou, China; ^2^ Institute of Statistics and Applications, Fujian Agriculture and Forestry University, Fuzhou, China

**Keywords:** *solanum melongena* L, anthocyanin, transcriptomic, metabolomic, MYB

## Abstract

**Introduction:**

Purple eggplant (*Solanum melongena* L.) is valued for its high anthocyanin content, yet the regulatory mechanisms driving anthocyanin biosynthesis during fruit development remain poorly understood. This study aimed to elucidate the dynamics of anthocyanin accumulation and identify key regulatory genes across three developmental stages of eggplant fruit peel (S1: white, S2: light purple, S3: dark purple).

**Methods:**

Integrated metabolomic and transcriptomic analyses were conducted to investigate anthocyanin profiles and gene expression patterns during fruit development. Metabolomic profiling identified anthocyanin types, while transcriptomic data highlighted differentially expressed genes. Transient overexpression assays of candidate MYB transcription factors (TFs) were performed to validate their regulatory roles in anthocyanin biosynthesis.

**Results:**

Total anthocyanin content significantly increased as the fruit peel color deepened from S1 to S3. Metabolomic analysis detected 11 distinct anthocyanins, with concentrations rising progressively across stages. Transcriptomics revealed 8 structural genes and 11 MYB TFs enriched in the anthocyanin pathway. Transient overexpression of *SmMYB32* and *SmMYB67* upregulated key biosynthetic genes (e.g., *CHS*, *DFR*, *ANS*), confirming their role in enhancing anthocyanin production.

**Discussion:**

This study demonstrates that anthocyanin accumulation in eggplant fruit peels is tightly linked to the upregulation of biosynthetic genes and MYB TFs during development. The functional validation of *SmMYB32* and *SmMYB67* highlights their potential as targets for breeding high-anthocyanin cultivars. These findings advance our understanding of anthocyanin regulation in eggplant and provide actionable strategies for improving nutritional quality through molecular breeding.

## 1 Introduction

Eggplant *(Solanum melongena* L.) is widely cultivated worldwide as an important crop (Cericola et al., 2014). The fruit of eggplants comes in various colors, including black, purple, red, lavender, white, and orange. Among these colors, purple eggplant is highly favored due to its rich anthocyanin content (Zhang et al., 2014), which has been associated with a reduced risk of chronic diseases such as cancer (Butelli et al., 2008; Guarnieri et al., 2007; Paredes-López et al., 2010; Pascual-Teresa et al., 2010; Toufektsian et al., 2008). Given the significant market demand and commercial interests involved, it is essential to further understand the accumulation of these protective compounds in purple eggplants.

Anthocyanins, which belong to flavonoids, play a crucial role in determining fruit color due to their distinctive molecular structures and high solubility in water. They can be classified into six groups based on variations in their molecular structures: malvidin, petunidin, pelargonidin, peonidin, delphinidin, and cyanidin. Their synthesis is regulated by various structural genes and regulatory genes through the flavonoid pathway (He and Giusti, 2010; Wei et al., 2011). Key structural genes involved in this process include chalcone synthase (*CHS*), chalcone isomerase (*CHI*), flavonol 3′-hydroxylase (*F3′H*), dihydroflavonol-4-reductase (*DFR*), uridine diphosphate (UDP)-glucose: flavonoid 3-O-glycosyltransferase (*UFGT*), and glutathione S-transferase (*GST*) (Jiang et al., 2016). Additionally, other TFs such as MYB (Dubos et al., 2010), WRKY (Li et al., 2020), NAC (Li et al., 2020), bZIP (An et al., 2017), and MADS (Jaakola et al., 2010), have also been proven to participate in regulating anthocyanin biosynthesis.

Understanding the mechanisms underlying phenotypic changes associated with anthocyanin accumulation requires uncovering the complex connections between genes and metabolites. Co-analysis techniques of transcriptome and metabolome can explore this relationship, providing a more accurate method to reflect these changes (Hirasawa et al., 2018; Robin et al., 2018). This approach has been widely used to identify signal pathway and mechanisms controlling changes in plant color during anthocyanin accumulationl. For example, Chen et al. (2021) conducted metabolomic and transcriptomic analyses on the young and old skins of cucumber color mutants L19 and near-isogenic line L14, revealing that four key transcription factors—R2R3-MYB, bHLH51, and WRKY23—are likely the main driving factors causing transcriptional differences in the peels of L14 and L19. Shi et al. (2020) analyzed the metabolomic and transcriptomic data of fruit skins at different developmental stages, finding that anthocyanin content was closely related to the expressions of *ZjANS* and *ZjUGT79B1* and their regulatory transcription factors ZjMYB5, ZjTT8, and ZjWDR3. Jia et al. (2020) performed transcriptomic and metabolomic analyses on apple red blushed-skin mutants (CF-B1 and CF-B2) and red striped-skin parents (CF-S1 and CF-S2) during the coloring period on the 4th, 6th, and 8th days after bag removal, identifying that apple anthocyanin biosynthesis is regulated by *MdMYB10*, *MdbHLH3*, and *MdWD40*. In eggplants, Li et al. (2023) conducted transcriptomic analyses on six eggplant varieties with different peel colors and two purple-peel transgenic eggplants overexpressing *SmMYB113*, identifying 27 new genes related to the differences in eggplant peel color and 32 new genes regulated by *SmMYB113* and involved in anthocyanin biosynthesis. Yang et al. (2022) conducted a metabolomic analysis on six different varieties of eggplants with varying peel colors at maturity to explore the relationship between anthocyanin composition and color. However, the current studies concentrate on mature fruits or specific time points, neglecting a comprehensive analysis of the dynamic regulatory network for anthocyanin synthesis and the fluctuations in metabolite fluxes across different developmental stages of eggplants.

This study aims to investigate the dynamic accumulation of anthocyanins in eggplant pericarp during fruit development. It will involve observing phenotypic color changes at three different stages and measuring the content of anthocyanin accumulation. Additionally, metabolomic and transcriptomic analyses will be conducted to explore variations in metabolites and genes related to anthocyanin during eggplant development. The findings from this research will provide insights into the mechanisms underlying the dynamic accumulation of anthocyanins in eggplant peel, thereby laying a foundation for genetic improvement and cultivation of new varieties that promote consumer health.

## 2 Materials and methods

### 2.1 Plant materials and sampling

The study was conducted at the Qishan campus experimental base of Fujian Agriculture and Forestry University. Eggplant pericarps were collected at three distinct development stages: 5 days after fruit set (young fruit stages, white coloration), 10 days after fruit set (Commodity maturity stage, light purple coloration), and 15 days after fruit set (physiological maturity stage, dark purple coloration). These peel samples were selected for total anthocyanin quantification, RNA-seq, and metabolomic analysis. Fresh eggplant fruits were carefully peeled using a razor blade and immediately frozen in liquid nitrogen before being stored at −80°C. For each developmental stage, samples were collected with three biological replicates, each consisting of peels from a minimum of five eggplants.

### 2.2 Quantification of the total anthocyanin aontent

Anthocyanins were extracted from a 0.05g fresh sample of eggplant pericarp and quantified using the method described by Niu et al. (2010). The total anthocyanin content was calculated as TA = A × MW × 5 × 100 × V/e, where TA represents the total anthocyanin content in milligrams per 100 grams, V is the final volume in milliliters, and A = [A510 nm (pH 1.0) – A700 nm (pH 1.0)] – [A510 nm (pH 4.5) – A700 nm (pH 4.5)] (Romero et al., 2008).

### 2.3 Metabolic analysis

Dry sample extraction, ultrahigh performance liquid chromatog raphy and tandem mass spectrometry, and the experimental data analysis were performed according to the methods of Wang et al. (2021). Metabolic data of each sample were analyzed by Principal component analysis (PCA), hierarchical cluster analysis (HCA), and K-means. The variables of the original state metabolomics data were represented by PCA. The HCA results of metabolites and samples are shown using heat maps with tree plots. HCA and PCA analyses were performed by Software R and GraphPad Prism v9.01 (GraphPad Software Inc., La Jolla, CA, USA), respectively. The raw data for the orthogonal partial least squares discriminant analysis (OPLS-DA) model underwent log_2_ transformation followed by mean centering to facilitate the identification of differential metabolites. Since the variable importance in projection (VIP) value indicates the impact of metabolites on the inter-group differences in sample classification, we established the screening criteria as follows: metabolites with a VIP > 1 and an absolute Log_2_FC (fold change) ≥ 1 were considered as significant differentially expressed metabolites (DEMs) (Yan et al., 2019). The venn diagram showed the quantity relationship of different comparison groups. The Kyoto Encyclopedia of Genes and Genomes (KEGG) compound database (https://www.kegg.jp/3eg/compound/) was used to annotate the different metabolites, which were mapped to the KEGG pathway database (https://www.kegg.jp/3eg/pathway.html).

### 2.4 Transcriptome analysis

The Total RNA Isolation Kit (Sangon, China) was utilized to extract total RNA from nine samples in accordance with the manufacturer’s instructions. Biomarker Biotechnology Corporation (Beijing, China) conducted library construction and RNA sequencing (RNA-seq) assay using standardized protocols that were optimized based on a method described by Liang et al. (2022). After removing reads containing adapter, poly-N, and low-quality sequences, the remaining clean reads were aligned to the reference genome of eggplant (http://eggplant-hq.cn). The aligned reads were then assembled and quantitatively analyzed using StringTie software to determine fragments per kilobase of exon per million fragments mapped (FPKM) values. Significant differentially expressed genes (DEGs) analysis was performed using DESeq software to compare the control group with the treatment groups (S1 *vs*. S2 and S1 *vs.* S3). We identified DEGs based on a false discovery rate (FDR) ≤ 0.05 and |log_2_FC| ≥ 1. Additionally, significant comparisons underwent KEGG enrichment analyses.

### 2.5 Subcellular localization, protein expression and transient expression analysis of candidate MYB TFs associated with anthocyanin synthesis

All primers used for gene cloning and plasmid construction are listed in **Supplementary Table S1**. For the construction of the plasmids used for the subcellular localization assays, the target genes were subsequently cloned and inserted into pSuper1300-GFP. The construction of the plasmids used for transient expression assays was reference Chen et al. (2021). All the plasmids generated in this study were verified by sequencing. *Agrobacterium tumefaciens* GV3101 was used for transient protein expression assays in *N. benthamiana* and *Solanum melongena* leaves. For the immunoblot assays, A. tumefaciens GV1301 cells at an OD600 of 0.6 were used for fusion protein expression, and agroinfiltrated plants were maintained under normal growth conditions for 48 to 72 h. For protein analysis, total proteins were extracted from *Solanum melongena* leaves was. Then, total protein was mixed with 2x Laemmli buffer at a ratio of 1:1, boiled for 10 min, and separated by SDS–PAGE for immunoblot analysis with the indicated antibodies. Confocal imaging was performed using a Leica SP8 X inverted confocal microscope with an argon laser (Leica, Wetzlar, Germany). The RFP fluorophore was excited at 561 nm, and the emission was captured at 490–542 nm. Images were captured digitally and processed using Leica Application Suite Advanced Fluorescence Lite software (Leica Microsystems). For transient expression experiments, the young and tender leaves of eggplant seedlings were selected for injection. Leaf samples were collected 72 hours post-injection from both the control and injected groups of eggplants. A minimum of three eggplant leaves per group were selected, with three biological replicates established for each sample. The collected samples were immediately frozen in liquid nitrogen and subsequently stored at -80°C for future use. RNA extraction and cDNA synthesis were performed for both the control and experimental groups, followed by the detection of relevant gene expression levels using qPCR.

### 2.6 Quantitative real-time PCR analysis

For qRT-PCR analysis, we selected structural genes and MYB TFs involved in anthocyanin biosynthesis. Specific primers for qRT-PCR were designed using Primer Premier v. 5.0 (Premier Biosoft, Palo Alto, CA, USA; **Supplementary Table S1**). The cDNA samples were assessed by qRT-PCR using SYBR Premix ExTaq (Takara). The relative expressions of mRNA in each sample were calculated by comparing them to the reference gene (Actin) using the comparative Ct (2^-ΔΔCt^) method. (Livak and Schmittgen, 2001) Finally, to validate the sequencing results’ reliability, we compared gene expression trends in stages S1, S2 and S3 between qRT-PCR and RNA-seq analyses.

## 3 Results

### 3.1 Effect of growth and development on the total anthocyanin content in purple eggplant peels

**Figure 1:**
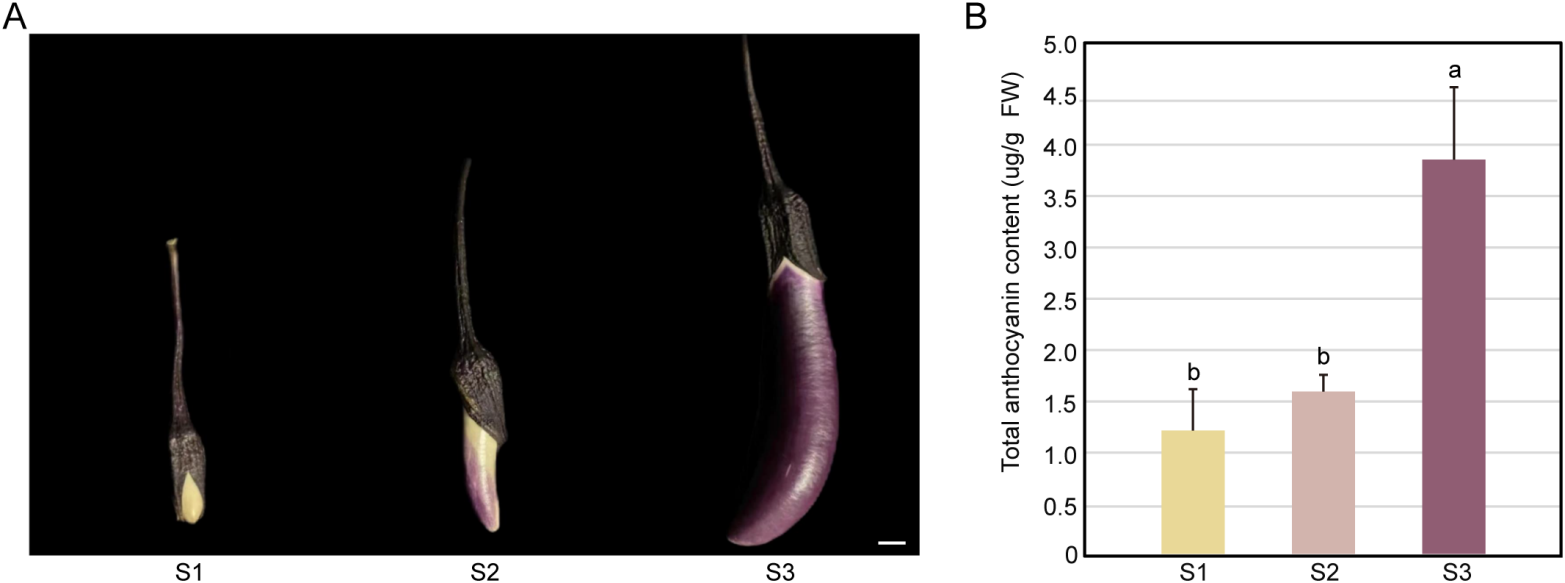
Comparative analysis of fruit morphology **(A)** and anthocyanin content **(B)** in purple eggplant fruits at three stages. Fruit setting stage – S1, rapid growth stage – S2, and commercial maturity stage – S3 occur 5 days, 10 days, and 15 days after fruit set in purple eggplants, respectively. The values represent the means of three independent experiments, with error bars indicating the standard deviations of three biological replicates. Note: Different lowercase letters in the figure indicate significant differences between treatments (*P* < 0.05).

Throughout the growth and development process, the color of eggplant peels undergoes a gradual transformation from white (S1) to light purple (S2), eventually reaching dark purple (S3; **Figure 1A**). Moreover, as the purple hue emerges and intensifies, there is a corresponding increase in anthocyanin content (**Figure 1B**), with anthocyanin levels at the three stages being 1.2 ug/g (S1), 1.5 ug/g (S2), and 3.8 ug/g (S3) respectively. These findings suggest that changes in eggplant peel color are linked to variations in anthocyanin content.

### 3.2 Metabolic profiling

**Figure 2:**
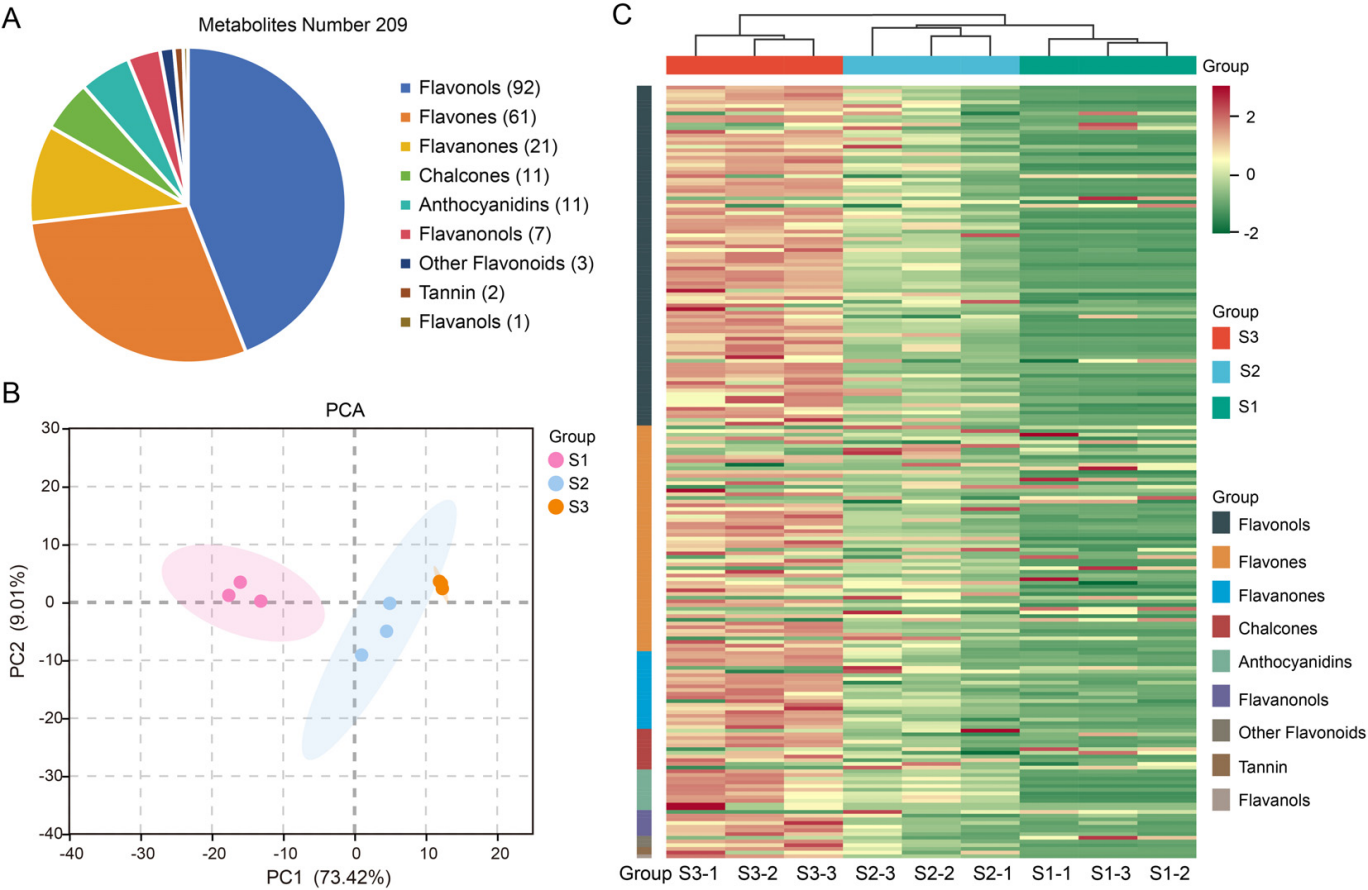
Analysis of flavonoid metabolites in the peel of purple eggplant during three growth stages. **(A)** Classification of 209 metabolites in purple eggplant fruit samples. **(B)** PCA plot of metabolomic data from the nine samples. **(C)** Cluster heat map of 209 metabolites.

To gain insights into the flavonoid metabolites present in different growth stages of purple eggplant fruit peel, we conducted a targeted metabolomic analysis. The pie chart (**Figure 2A**) revealed a total of 209 identified flavonoid metabolites, including 92 flavonals, 61 flavones, 21 flavanones, 11 chalcones, 11 anthocyanidins, 7 flavanonols, 2 tannins, 1 flavanols and 3 other flavonoids. PCA showed that the first two principal components (PC1 and PC2) explained 73.42% and 9.01% of the total variance, respectively (**Figure 2B**). Both PCA and HCA (**Figure 2C**) indicated that while the S2 and S3 groups exhibited higher similarity compared to the S1 group, all three groups were distinctly separated, suggesting substantial variation in metabolite levels across different growth stages.

**Figure 3:**
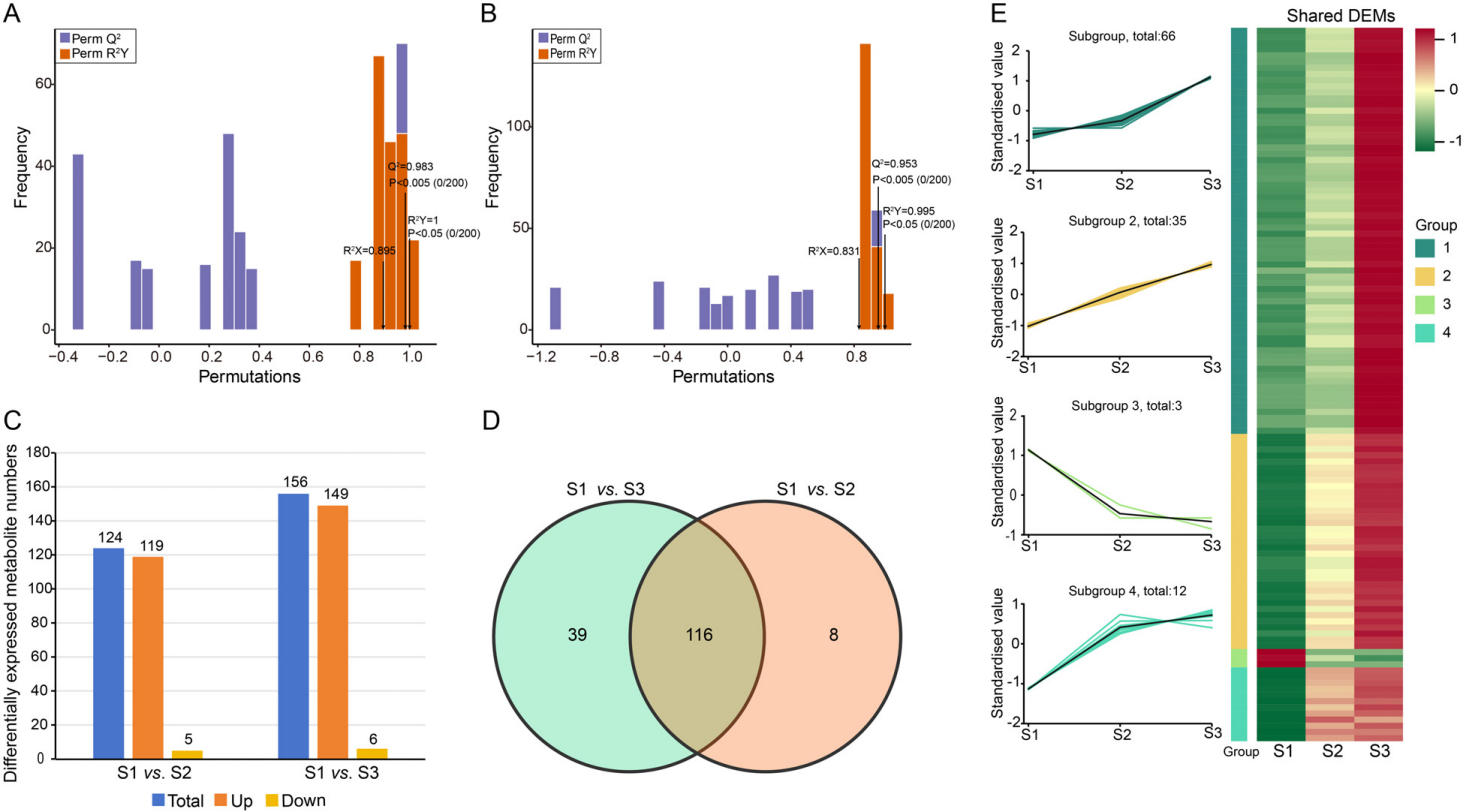
DEMs of two comparison groups (S1 *vs*. S2, S1 *vs*. S3). The score charts of the OPLS-DA model for S1 *vs*.S2 **(A)** and S1 *vs*. S3 **(B)**. **(C)** Bar graph of DEMs for the two comparison group. **(D)** Venn diagram showing the numbers of common and specific DEMs among the different comparisons. **(E)** Line charts plot of K-means clustering of DEMs and visualization of clustering heat map.

Based on the OPLS-DA detection model with Q^2^ > 0.9, we identified DEMs between two comparison groups (**Figures 3A, B**). The analysis of all DEMs based on bar charts revealed a total of 124 DEMs detected in the S1 *vs.* S2 comparison group, with 119 up-regulated and five down-regulated. In the S1 *vs.* S3 comparison group, there were 155 detected metabolites, with 149 significantly up-regulated and six down-regulated (**Figure 3C**; **Supplementary Table S2**). Furthermore, Venn diagrams showed that there were a total of 116 shared metabolite species between the two comparison groups (**Figure 3D**). K-means clustering analysis demonstrated that these116 DEMs could be divided into four clusters (**Figure 3E**). The relative abundance of each cluster’s metabolites exhibited similarity within their respective clusters. Notably, cluster one contained the largest number of metabolites (66), followed by cluster two (35) and cluster four (12), while cluster three had only three included metabolites. Additionally, during eggplant peel coloration process, the metabolites included in clusters one, two and four showed an increasing trend whereas those in cluster three exhibited a decreasing trend.

### 3.3 Transcriptome sequencing

To investigate the impact of different growth and development stages on the transcriptome of purple eggplant, we prepared nine cDNA libraries for RNA-seq analysis. After conducting rigorous quality control on the sequencing data, we obtained a total of 59.45 Gb of data, including 3.96 million paired-end sequence reads. The Q30 ratio exceeded 93.48%, and the GC content ranged from 41.15% to 42.44% (**Table 1**). The percentage of transcriptome sequencing data aligned to the eggplant reference genome was between 90.31% and 93.08%.

**Table 1:** Summary of the sequence analysis of nine libraries.

Sample	Clean Reads	Clean Base(G)	Q30(%)	GC Content (%)	Unique mapped
S1-1	43,272,376	6.49	93.69	42.44	39,079,030(90.31%)
S1-2	42,991,188	6.45	93.68	42.50	39,317,975 (91.46%)
S1-3	45,529,850	6.83	93.88	41.83	41,509,301 (91.17%)
S2-1	40,664,504	6.10	93.90	41.28	38,541,971 (93.08%)
S2-2	46,397,212	6.96	93.48	41.15	41,911,076 (91.66%)
S2-3	45,745,368	6.86	93.66	41.47	41,071,548 (92.11%)
S3-1	41,409,022	6.21	93.89	41.62	37,818,962 (93.00%)
S3-2	45,726,084	6.86	93.78	41.40	43,150,247 (93.00%)
S3-3	44,588,084	6.69	93.91	41.62	42,546,886 (93.01%)

### 3.4 Analysis and functional annotation of DEGs

**Figure 4:**
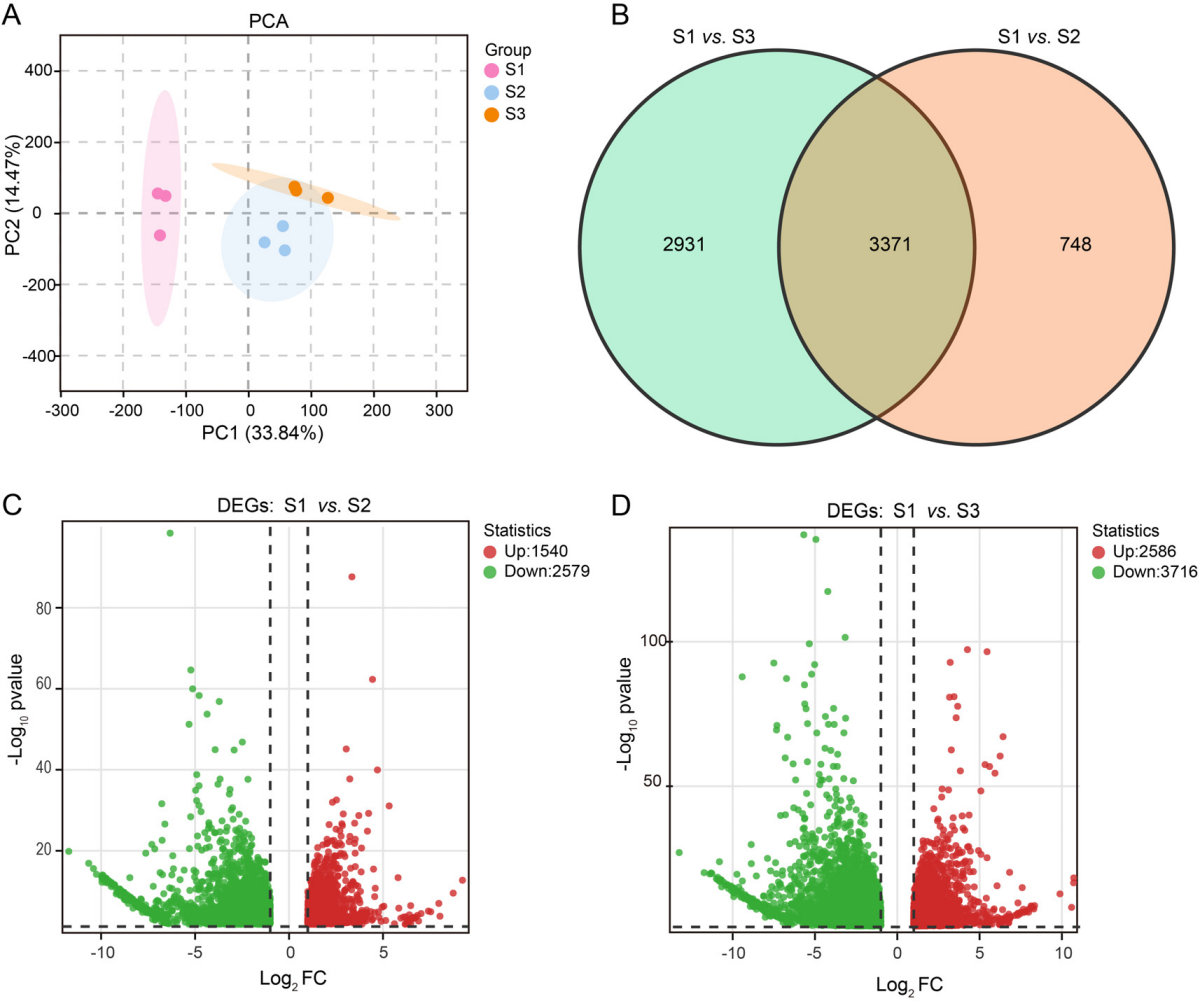
Analysis of transcriptome differences between the S1 and S2 groups and between the S1 and S3 groups. **(A)** PCA analysis of transcriptome data of 9 samples. **(B)** The venn diagram showing specific and common DEGs. The non-overlapping area of the Venn diagram represents the DEGs specific to the subgroup comparison, and the overlapping area represents the DEGs common to the several subgroup comparisons. Volcano plots of the DEGs for S1 *vs.* S2 **(C)** and S1 *vs.* S3 **(D)**. Each point in the volcano map represents a gene, with green and red points representing down-regulated and up-regulated genes, respectively.

PCA analysis (**Figure 4A**) demonstrated that samples within each group exhibited clustering, indicating high repeatability in the transcriptome analysis. Moreover, clear separation between different groups was observed, making it suitable for detecting group differences. Comparing the S1 and S2 groups as well as the S1 and S3 groups revealed a total of 7,050 DEGs. Among these, 3,371 expressed genes were shared in both the S1 *vs.* S2 and S1 *vs.* S3 comparisons (**Figure 4B**; **Supplementary Table S3**). When comparing the S1 and S2 groups, we identified 4,119 DEGs with 2,579 down-regulated and 1,540 up-regulated genes (**Figure 4C**). Furthermore, when comparing the S1 and S3 groups, we identified 6,302 DEGs with 3,716 down-regulated and 2,586 up-regulated genes (**Figure 4D**). These findings suggest that growth stages significantly influence gene expression in purple eggplant.

### 3.5 DEMs and DEGs related to anthocyanin biosynthesis

**Figure 5:**
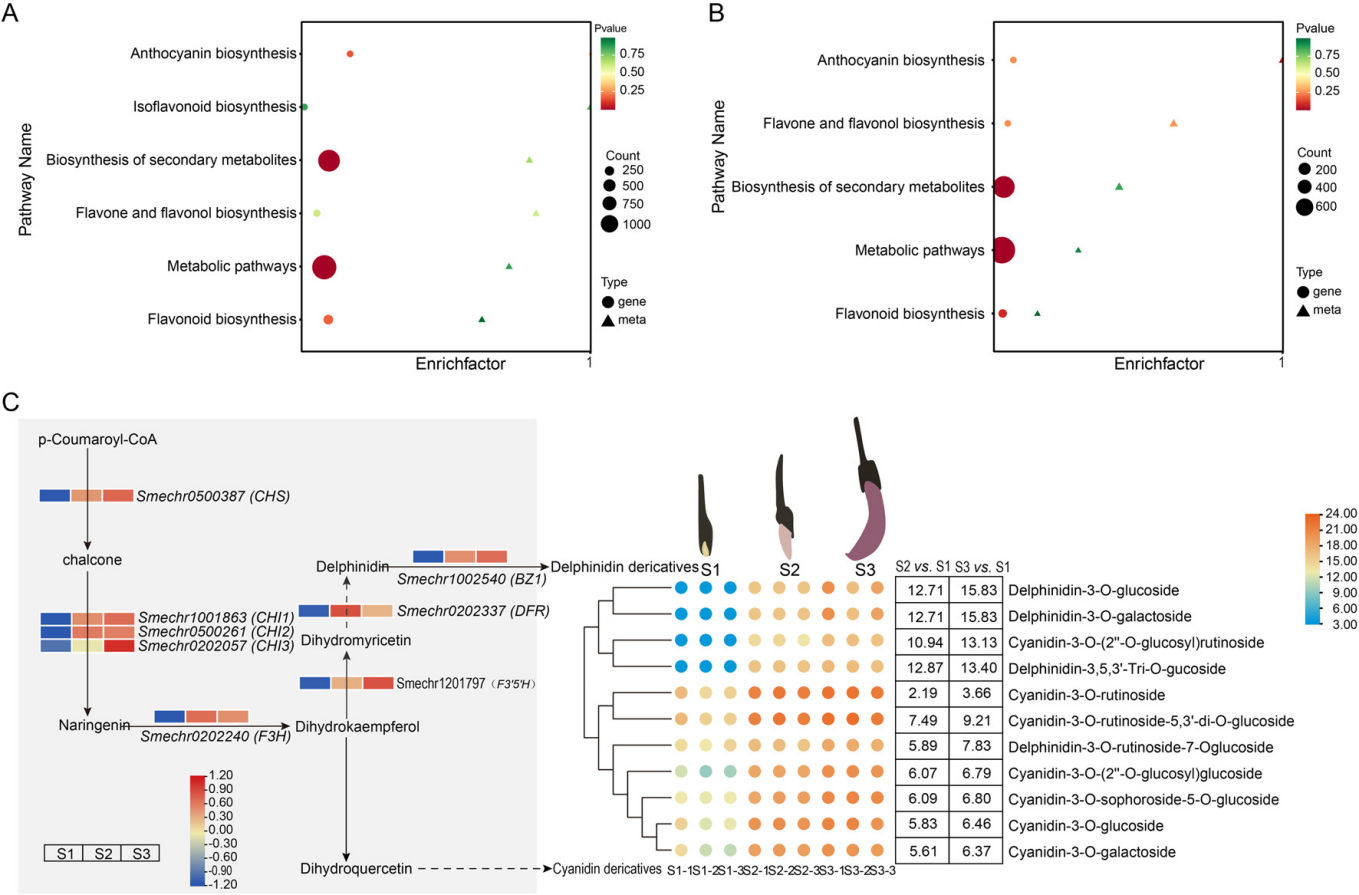
KEGG analysis of DEGs and DEMs. KEGG enrichment bubble plots based on S1 *vs.* S2 **(A)** and S1 *vs.* S3 **(B)**. The x-axis represents the enrichment factor of the pathways (Diff/Background), and the y-axis represents the names of the KEGG pathways. The gradient from red to yellow to green indicates the significance of enrichment, with P-values representing the degree of significance. The shape of the bubbles corresponds to different omics types, while the size of the bubbles reflects the number of differentially expressed metabolites or genes, with larger bubbles indicating a greater number. **(C)** Changes in metabolites and genes related to the anthocyanin biosynthesis pathway during the growth and development of eggplant. FPKM values for eight structural genes were transformed by log_2_(n+1), n = FPKM values. The numbers in the box indicate the Log2 (FC) (S1 *vs.* S2 and S1 *vs.* S3) of metabolites in eggplant. Note: KEGG rich analysis was performed using the Metware Cloud, a free online platform for data analysis (https://cloud.metware.cn).

KEGG analysis of DEGs and DEMs between the S1 and S2 and between the S1 and S3 groups demonstrated that these genes and metabolites were highly enriched in anthocyanin synthesis (Ko00942; **Figures 5A, B**), indicating that the expression level of anthocyanin biosynthesis genes and metabolites is affected by growth and development, with notable differences observed across the three different developmental stages.

When comparing between the S1 and S2 groups and between the S1 and S3 groups, we identified eight structural DEGs involved in the anthocyanin biosynthesis pathway (**Figure 5C**). These included one *CHS* gene, three *CHI* genes, one *F3’5H* gene, one *F3H* gene, one *DFR* gene, and one *BZ1* gene, all of which were up-regulated.

Eleven anthocyanins including seven cyanidins and four delphinidins were detected in the peels of purple eggplant (**Figure 5C**). All the 11 anthocyanins were significantly upregulated in the S2 and S3 groups compared with the S1 control group. Four anthocyanins, namely delphinidin-3-O-glucoside, delphinidin-3-O-galactoside, and cyanidin-3-O-(2”-o-glucosyl) rutinoside, exhibited major changes. When comparing between the S1 and S2 groups, the change values of log_2_ (FC) were 12.71, 12.71, 10.94, and 12.87, respectively. Furthermore, when comparing between the S1 and S3 groups, the change values of log_2_ (FC) were 15.83, 15.83, 13.13, and 13.40, respectively. Seven anthocyanins, namely cyanidin-3-O-rutinoside, cyanidin-3-O-rutinoside-5, 3’-di-O-glucoside, delphinidin-3-O-rutinoside-7-O-glucoside, cyanidin-3-O-(2’’-O-glucosyl) glucoside, cyanidin-3-O-sophoroside-5-O-glucoside, cyanidin-3-O-glucoside, and cyanidin-3-O-galactoside, displayed relatively minor changes. These results indicate that cyanidins and delphinidins were closely associated with color changes in purple eggplant at different developmental stages.

### 3.6 TFs analysis

**Figure 6:**
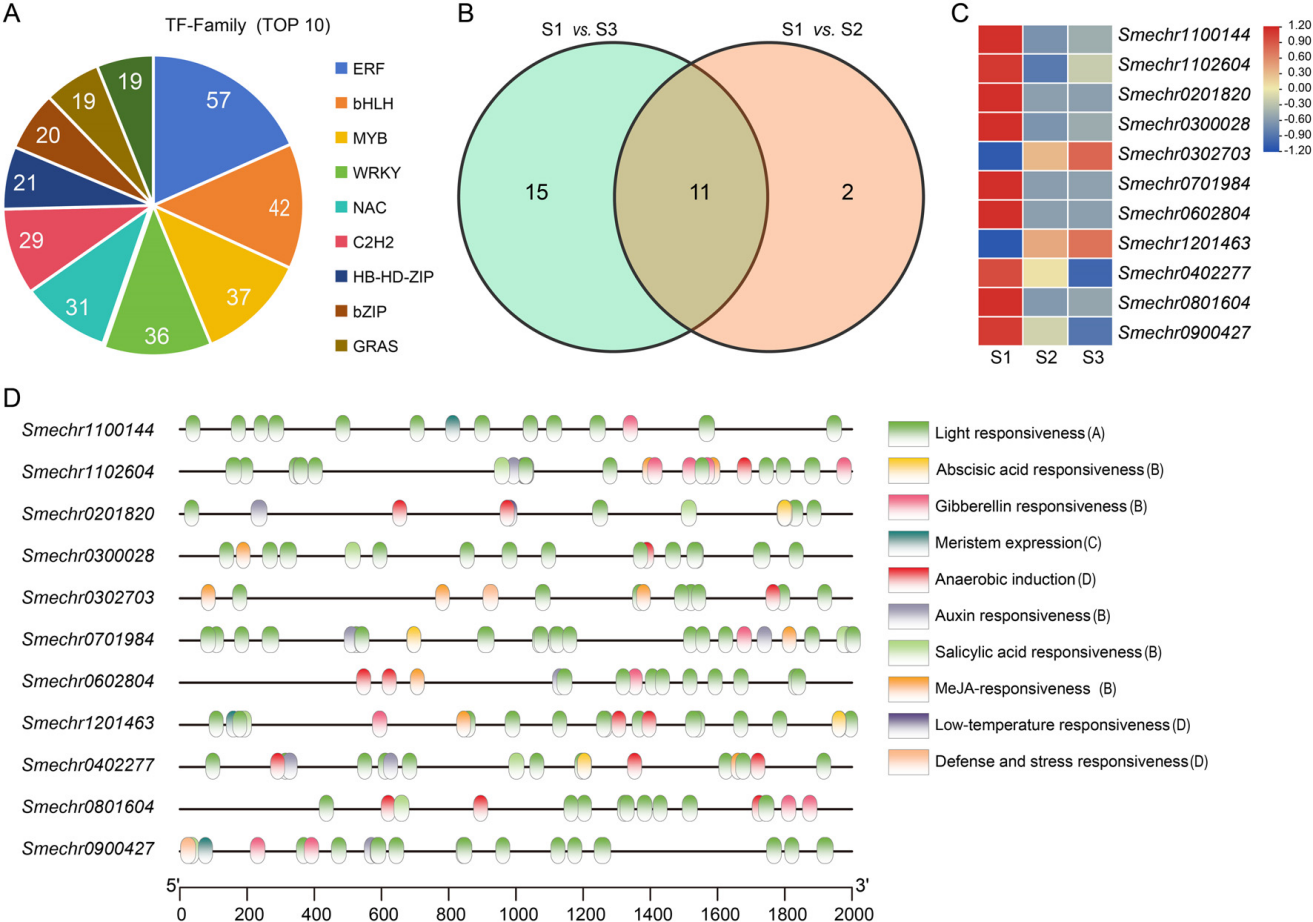
TFs of purple eggplant. **(A)** The pie chart displays the top10 TFs. **(B)** Veen diagrams showing specific and common differentially expressed *MYBs* in S1 *vs.* S2 and S1 *vs.* S3. **(C)** Heat map illustrates the expression level changes of 11common *MYBs* during the three developmental stages of eggplant fruit. FPKM values for *MYBs* were transformed by log_2_(n+1), n = FPKM values. **(D)** Analysis of cis-acting elements for the common *MYBs*. Note: **(A)**: Photoresponsive cis-acting element. **(B)**: Hormone response-related cis-acting elements. **(C)**: Growth and development related cis-acting element. **(D)**: cis-acting elements associated with stress.

During the fruit skin coloration process in eggplant, TFs play a critical role in modulating the expression of target genes. The pie chart illustrates the top 10 TFs, revealing that the ERFs are the most abundant with 57, followed by bHLH (42), MYB (37), and WRKY (36). GRASs and bZIPs are relatively less represented, each containing 19 (**Figure 6A**).

Among these, *MYBs* are particularly significant as key regulators in the direct control of anthocyanin synthetase expression. Consequently, *MYBs* have emerged as pivotal TFs in the investigation of the anthocyanin biosynthetic pathway. In the present study, 13 and 26 differentially expressed *MYBs* were identified in comparisons between the S1 and S2 groups and between the S1 and S3 groups, respectively. Notably, 11 *MYBs* were consistently expressed across both comparison groups (**Figure 6B**). Analysis of the expression patterns of these 11 common *MYBs* during the three developmental stages of eggplant fruit skin (**Figure 6C**) revealed a predominant decreasing trend in expression levels for the majority of *MYBs* (9 out of 11) across S1, S2, and S3 stages. Conversely, two *MYBs* (*Smchr0302703* and *Smechr1201463*) exhibited an increasing expression trend. Furthermore, the promoter regions of these *MYBs* were found to harbor *cis*-elements associated with photoreception, hormonal responses, growth and development, and stress responses (**Figure 6D**), indicating that these *MYBs* may play a role in the modulation of peel coloration throughout the growth and development of eggplant.

### 3.7 Subcellular localization and expression analysis of *Smchr0302703(SmMYB32)* and Smchr1201463(SmMYB67)

**Figure 7:**
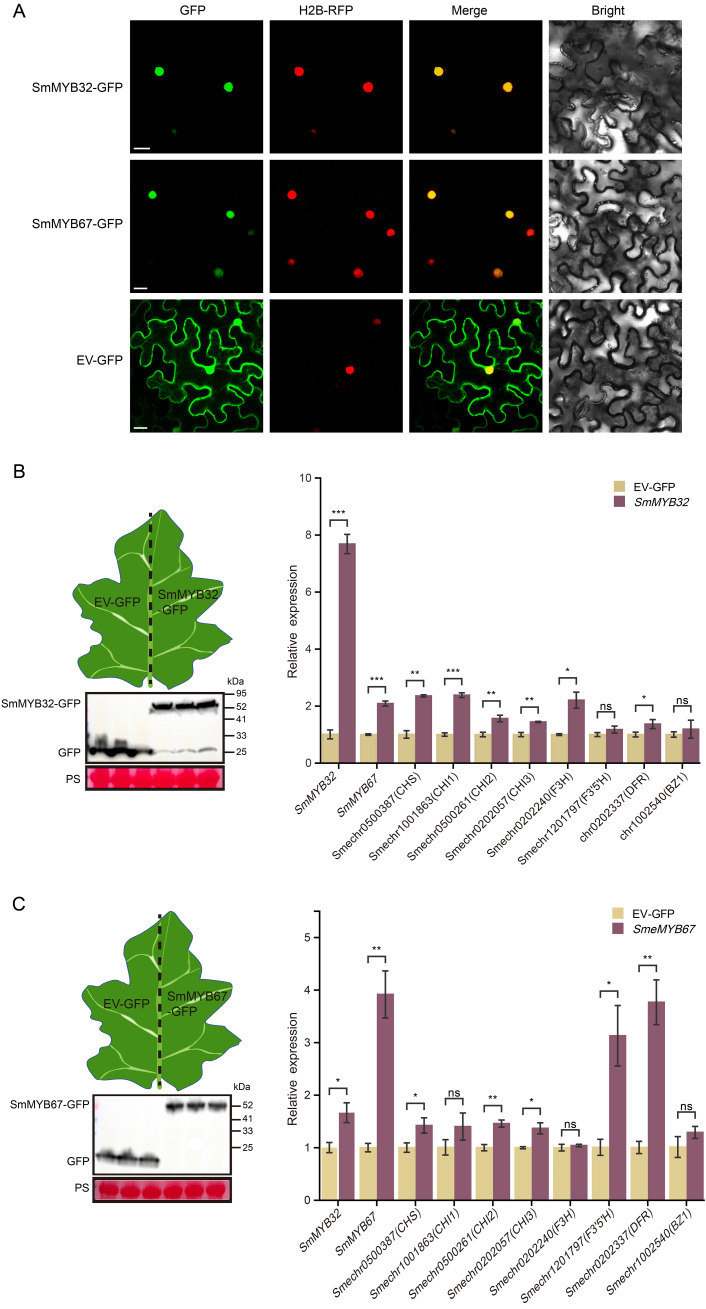
Subcellular localization and expression analysis of *Smchr0302703(SmMYB32)* and *Smchr0302703(SmMYB67).* **(A)** H2B-RFP was transiently co-expressed with SmMYB32-GFP, SmMYB67-GFP and EV-GFP in *N. benthamiana* leaves. Bar = 20 μm. **(B, C)** Western blotting analysis of EV-/SmMYB32-/SmMYB67-GFP in leaves of eggplant. Leaves were harvested at 3 days and total protein extracts were blotted against GFP antibody. PS staining shows equal protein loading. PS: Ponceau S. Expression analysis of anthocyanin biosynthesis marker genes in control (EV-GFP) and experimental group (SmMYB32-GFP and SmMYB67-GFP). The expression profiles of *SmMYB32*, *SmMYB67*, *CHS*, *CHI1*, *CHI3*, *F3H*, *F3’5’H*, *DFR*, and *BZ1* in the leaves of eggplant were analyzed following the transient overexpression of *SmMYB32*-GFP, *SmMYB67*-GFP, and EV-GFP. Error bars represent the SD of three independent experiments. Statistical significance was tested with t-test (*, *P* < 0.05; **, *P* < 0.01; ***, *P* < 0.001). NS, no significance.

To gain a deeper understanding of the roles of *Smchr0302703* and *Smechr1201463* in anthocyanin synthesis in eggplant fruit peels, we cloned these two genes. The open reading frames (ORFs) of *Smchr0302703* and *Smechr1201463* are 981 bp and 663 bp respectively, encoding proteins of 326 and 220 amino acids (**Supplementary Table S4**). Phylogenetic analysis with *MYB*s from eggplant and *Arabidopsis thaliana* led us to designated them as *SmMYB32* and *SmMYB67* (**Supplementary Figure S1**). To determine the subcellular localization of *SmMYB32* and *SmMYB67*, we performed confocal microscopy analysis in *Nicotiana benthamiana* leaves. Confocal microscopy analysis conducted 48 hours post-infiltration showed that both *SmMYB32*-GFP and SmMYB67-GFP co-localized with the nuclear marker H2B-RFP (**Figure 7A**).

To investigate the functional impact of *SmMYB32* and *SmMYB67* on anthocyanin biosynthesis, we transiently overexpressed these genes in eggplant leaves via *Agrobacterium*-mediated infiltration. The results showed no apparent accumulation of purple pigments on the leaf surface. Nevertheless, Western blot analysis confirmed the successful expression of these two genes. Concurrently, RT-qPCR analysis further demonstrated that overexpression of *SmMYB32* and *SmMYB67* significantly up-regulated anthocyanin biosynthesis-related genes, including *CHS*, *CHI1*, *CHI2*, *CHI3*, and *DFR*, three days post-agroinfiltration (**Figures 7B, C**). These data collectively establish the functional involvement of both MYBs in activating the anthocyanin pathway.

### 3.8 QRT-PCR validation

**Figure 8:**
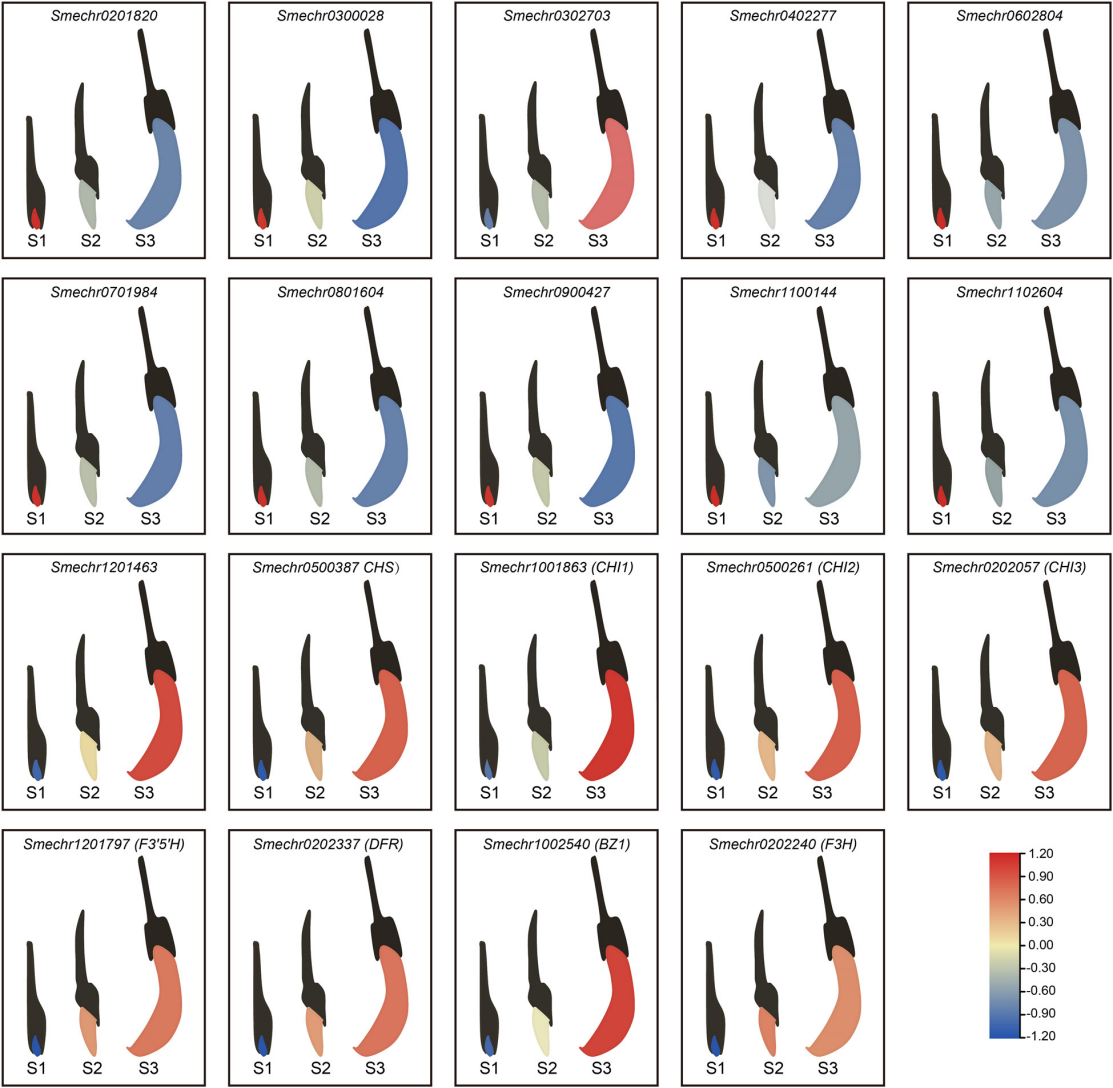
Expression patterns of 19 DEGs associated with anthocyanin biosynthesis. The gene expression levels in different tissues of the eggplant are represented by varying colors. The color blue indicates lower gene expression, while red indicates higher expression levels. All expression levels are calculated as the average of three replicates. The expression levels of all genes have been scaled in three stages.

To verify the reliability of the transcriptome data, we analyzed 19 DEGs (8 structural genes and 11 MYB TFs) associated with anthocyanin biosynthesis through real-time PCR (qRT-PCR). Compared with the S1 group, both the S2 and S3 groups exhibited 10 up-regulated genes and 9 down-regulated genes. Among the up-regulated genes, eight (*Smechr0500387*, *Smechr1001863*, *Smechr0500261*, *Smechr0202057*, *Smechr1201797*, *Smechr0202337*, *Smechr1002540*, and *Smechr0202240*) were structural genes. The expression patterns of these 19 genes, as revealed by RNA-seq (**Figures 5C**, **6C**), were highly consistent with those obtained through qRT-PCR (**Figure 8**). These results demonstrated the high reliability of the transcriptome-based DEG analysis.

## 4 Discussion

Anthocyanins and their derivatives are crucial coloring substances, offering a wide range of colors from orange/red to violet/blue (Naing and Kim, 2021). They contribute to color expression in various fruits and crops. In this study, we quantified and identified anthocyanin contents in the peel samples collected during the S1, S2, and S3 stages (**Figure 1B**). The anthocyanin content increased with fruit development, being highest in the S3 stage, followed by the S2 and S1 stages. These results indicated that higher anthocyanin contents corresponded to a deeper purple peel color, aligning with the findings reported by Yang et al. (2022) for eggplants with different peel colors and those of Lai et al. (2016) for purple leaves of the novel tea cultivar “Ziyan”

Targeted metabolomics analysis can help identify specific metabolites responsible for color expression. A previous study reported that cyanidin 3-O-glucoside is the main anthocyanin in blood-flesh peaches (Yan et al., 2017). Additionally, six acylated pelargonidin 3-O-sambubioside-5-O-glucosides were isolated from the red-purple flowers of *Lobularia maritima* (Tatsuzawa et al., 2010). Our targeted metabolomics analysis in eggplant (**Figure 5C**) identified 11 anthocyanins in the peel, all belong to cyanidin and delphinidin anthocyanins. Previous studies have also reported that delphinidins are the major anthocyanins in eggplants with purple peels (Lazr et al., 2021; Yang et al., 2022).

The determination of total anthocyanin content (**Figure 1B**) and targeted metabolomics analysis (**Figure 5C**) revealed that during the S1 stage, despite some anthocyanin accumulation, its low level failed to reach the coloration threshold. Consequently, the white epidermis of eggplants did not exhibit purple pigmentation. In the S2 stage, although no significant statistical difference in total anthocyanin content was observed compared with the S1 stage (**Figure 1B**), transcriptomic analysis demonstrated that genes related to anthocyanin synthesis, such as *CHS*, *CHI*, and *F3H*, were significantly upregulated. Moreover, targeted metabolomics identified 11 anthocyanin derivatives that showed a significant increase in the S1 *vs.* S2 group. These derivatives may establish a positive regulatory loop, further promoting anthocyanin accumulation and enabling it to surpass the coloration threshold, thereby causing the eggplant peel to appear purple in the S2 stage. This mechanism is analogous to the coloration process driven by cyanidin derivatives reported in studies on RP longan (Yi et al., 2021) and purple grains (Wang et al., 2021).

In the PCA (**Figures 2B**, **4A**), the S2 and S3 stages showed greater similarity, consistent with the observation that both stages displayed a purple phenotype. The deeper coloration in the S3 stage compared to S2 is attributed to the continuous accumulation of anthocyanins during these growth periods. Further investigation into DEGs (**Supplementary Table S5**) and DEMs (**Supplementary Table S5**) revealed only 539 DEGs between S2 and S3, significantly fewer than in other comparisons (S1 *vs.* S2 and S1 *vs.* S3). Additionally, differential metabolite analysis indicated that only four out of eleven anthocyanins showed a significant increase from S2 to S3, including Cyanidin-3-O-rutinoside, Delphinidin-3-O-rutinoside-7-O-glucoside, Cyanidin-3-O-rutinoside-5,3’-di-O-glucoside, and Cyanidin-3-O-(2’’-O-glucosyl)rutinoside. However, highly glycosylated derivatives such as Cyanidin-3-O-(2’’-O-glucosyl)glucoside and Cyanidin-3-O-sophoroside-5-O-glucoside remained in dynamic equilibrium. These findings suggest that from S2 to S3, the focus shifts toward maintaining mature homeostasis rather than synthesizing new anthocyanin-related metabolites. This implies that later coloring depends more on vacuolar transport efficiency rather than *de novo* synthesis (Jaakola et al., 2010). Although the roles of transport proteins like GST or MATE were not directly validated in this study, their mechanisms could be elucidated through subcellular localization or gene silencing experiments in future research.

Changes in the accumulation of secondary metabolites correspond to changes in gene transcriptional abundance in the corresponding biosynthetic pathways. We combined the metabolomic and transcriptomic analyses to elucidate molecular mechanisms underlying the growth and development of purple eggplant through bioinformatics analysis (**Figures 4A, B**). KEGG enrichment analysis revealed that the anthocyanin biosynthesis pathway was the most active biological pathway for DEGs and DEMs when comparing between the S1 and S2 groups and between the S1 and S3 groups. By integrating transcriptomic and metabolomic analyses, we observed a significant increase in the expression levels of structural genes associated with anthocyanin synthesis during the S2 and S3 stages (**Figures 4C**). Specifically, these genes include *CHS* (*Smechr0500387*), *CHI* (*Smechr1001863*, *Smechr0500261*, and *Smechr0202057*), *F3H* (*Smechr0202240*), *F3’5’H* (*Smechr1201797*), *DFR* (*Smechr0202337*), and *BZ1* (*Smechr1002540*). The subsequent qRT-PCR results also confirmed this expression trend (**Figure 8**). This increased gene expression possibly accounts for the observed anthocyanin accumulation in the S2 and S3 stages based on pelargonidin and cyanidin compounds. Similar observations have been made in studies on the anthocyanin accumulation process in the red bark of *Ginnamomum camphora* (Zhong et al., 2022), red Chinese pear fruits (Ni et al., 2020) and rice purple endosperm (Zhu and Qian, 2017).

TFs that regulate anthocyanin biosynthesis have been identified in a diverse group of plants (Allan et al., 2008; Palapol et al., 2009), with MYBs being widely reported for their role in this process (Lu et al., 2019; Luo et al., 2017; Schwinn et al., 2019; Mondal and Roy, 2018; Zimmermann et al., 2004). In our study, 11 MYBs exhibited significant differential expression in both comparison groups (**Figure 6B**). Among these, *SmMYB32* and *SmMYB67* were notably upregulated, (**Figure 6C**). Further transient overexpression of *SmMYB32* and *SmMYB67* increased the expression of genes involved in the anthocyanin synthesis pathway, including *CHS*, *CHI*, *F3H*, *F3’5’H*, *DFR*, and *BZ1* (**Figures 7B, C**). These findings support the hypothesis that differentially expressed MYBs may act as candidate regulators of the anthocyanin synthesis pathway in eggplant. However, the cooperative regulation of MYBs with other TFs (e.g., bHLHs or WD40) remains unclear in eggplant. Further protein interaction assays (e.g., Y2H or BiFC) are required to dissect the regulatory complex driving anthocyanin accumulation.

## 5 Conclusion

In this study, we conducted transcriptomic and metabolomic analyses of eggplant at the S1, S2, and S3 stages, revealing that the purple pigmentation observed during the fruit development of purple eggplant is attributed to the synthesis of cyanidin and delphinidin. We identified eight DEGs involved in the anthocyanin biosynthesis pathway, with their expression levels significantly elevated during the S2 and S3 stages. This increase in gene expression likely correlates with the enhanced production of cyanidin and delphinidin. Additionally, we detected 11 MYB TFs that were differentially expressed between the S1 and S2 groups, as well as between the S1 and S3 groups, indicating their potential role as regulatory candidates in anthocyanin biosynthesis in eggplant. Our findings not only highlight key genes associated with the anthocyanin biosynthesis pathway throughout the growth and development of eggplant but also provide valuable insights into the mechanisms underlying fruit coloration across different growth stages.

## Data Availability

The datasets presented in this study can be found in online repositories. The raw reads were submitted to NCBI SRA (Sequence Read Archive, http://www.ncbi.nlm.nih.gov/sra/) under the accession number PRJNA1025879, while the generated datasets is provided within the manuscript or supplementary information files.
