# Supplementary figures and images for: Transcriptomic and metabolomic analyses for the mechanism underlying anthocyanin synthesis during the growth and development of purple eggplant (Solanum melongena L)
Source: Front Plant Sci. 2025 May 15;16:1577367. doi: 10.3389/fpls.2025.1577367 (PMC12119684; doi:10.3389/fpls.2025.1577367)

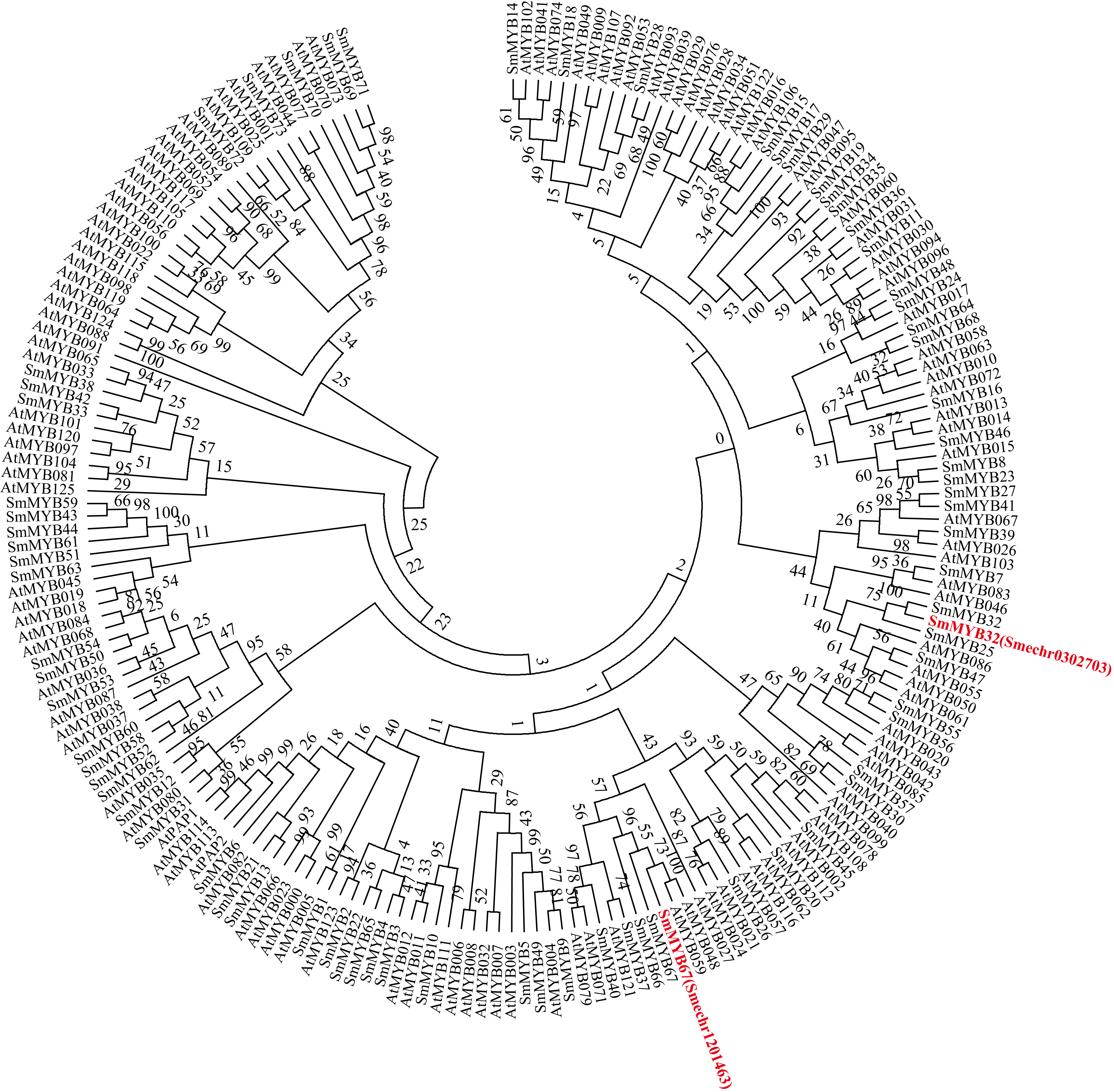

Supplement: Supplementary file 6 [file Image1.tif]
